# Increases in the autistic trait of attention to detail are associated with decreased multisensory temporal adaptation

**DOI:** 10.1038/s41598-017-14632-1

**Published:** 2017-10-30

**Authors:** Ryan A. Stevenson, Jennifer K. Toulmin, Ariana Youm, Richard M. A. Besney, Samantha E. Schulz, Morgan D. Barense, Susanne Ferber

**Affiliations:** 1 Western University, Department of Psychology, London, ON Canada; 2 Western University, Brain and Mind Institute, London, ON Canada; 3 Western University, Program in Neuroscience, London, ON Canada; 4 Western University, Department of Psychiatry, London, ON Canada; 5 York University, Centre for Vision Research, Toronto, ON Canada; 6 The University of Toronto, Department of Psychology, Toronto, ON Canada; 7 The Rotman Research Institute, Toronto, ON Canada

## Abstract

Recent empirical evidence suggests that autistic individuals perceive the world differently than their typically-developed peers. One theoretical account, the predictive coding hypothesis, posits that autistic individuals show a decreased reliance on previous perceptual experiences, which may relate to autism symptomatology. We tested this through a well-characterized, audiovisual statistical-learning paradigm in which typically-developed participants were first adapted to consistent temporal relationships between audiovisual stimulus pairs (audio-leading, synchronous, visual-leading) and then performed a simultaneity judgement task with audiovisual stimulus pairs varying in temporal offset from auditory-leading to visual-leading. Following exposure to the visual-leading adaptation phase, participants’ perception of synchrony was biased towards visual-leading presentations, reflecting the statistical regularities of their previously experienced environment. Importantly, the strength of adaptation was significantly related to the level of autistic traits that the participant exhibited, measured by the Autism Quotient (AQ). This was specific to the *Attention to Detail* subscale of the AQ that assesses the perceptual propensity to focus on fine-grain aspects of sensory input at the expense of more integrative perceptions. More severe *Attention to Detail* was related to weaker adaptation. These results support the predictive coding framework, and suggest that changes in sensory perception commonly reported in autism may contribute to autistic symptomatology.

## Introduction

Autism Spectrum Disorder (ASD) is a pervasive developmental disorder defined by two overarching characteristics: difficulties in social interactions and communication, and restricted interests and repetitive behaviours. With the release of the DSM-5^1^, atypical sensory processing was included as a diagnostic factor. This inclusion reflects a long known association between sensory processing disturbances and ASD, as well as more recent findings that sensory difficulties are in fact one of the most common characteristics of autistic individuals^2^. One area of sensory processing that has received considerable attention in autism is the ability to integrate sensory information across multiple input modalities^3^. Typically, when sensory information is processed through more than one modality, for example hearing a voice and seeing the facial articulation of a speaker’s mouth, this information is bound together. The result is a single, unified percept of the event – a process known as multisensory integration. Numerous recent reports suggest that this process may be compromised in autism, particularly as it pertains to the integration of auditory and visual social information^4–15^. Challenges related to multisensory integration in this population have also been theoretically^16^ and experimentally^17^ linked to higher-level cognitive processes that build on the processing of such bound sensory information.

Multisensory integration abilities are not static, but improve throughout typical development concomitantly with an individual’s exposure to the statistical regularities of the environment that predict whether two sensory inputs from different modalities originated from the same external event and thus should be integrated. One particular environmental cue to integrate is the temporal relationship of two sensory inputs: The more temporally proximate two sensory inputs are, the more likely they are to be integrated as one percept^18–31^, as shown repeatedly in samples of typically-developed observers. In typical development, audiovisual temporal perception has a protracted developmental course, however, with increases in multisensory temporal acuity observed into adolescence^20,32^. Autistic children and adolescents, on the other hand, exhibit decreased audiovisual temporal acuity relative to their typically-developed peers^4–6,17,33–40^. Such decreases in multisensory temporal acuity in autism have been directly linked to their impaired ability to integrate the auditory and visual signals that characterize speech in social interactions^6,17^.

We hypothesized that this concurrent decrease in multisensory temporal acuity and multisensory integration may be owing to a decreased ability to learn the statistical regularities in the environment. This can be conceptualized in the predictive coding framework. Importantly, this framework suggests that our mental representations of the world rely not only on current sensory information, but also on our past experiences. This has been formally defined using Bayesian statistics, where a weighted, generative model is built from inputs (current sensory information), and from an internal probability map based on prior experience^41,42^. Through this framework, it has been postulated that that autistic individuals may overly weight current sensory inputs relative to prior probabilities. This could arise in two ways. First, autistic individuals may develop a less robust probabilistic representation of the world^43–45^, i.e. a weak (flat) prior probability distribution^45^. Difficuluty creating a reliable probability map may would thus lead to problems understanding the likelihood of co-occurrence of events in the real world. Second, autistic individuals may learn appropriate prior probabilities, but more heavily weight incoming sensory information^46^. In these ways, perception differences in autism may arise from a decreased ability to compare current sensory information to an internal probabilistic “map” of the world^47^. Applied to the current topic, autistic individuals may have a decreased ability to learn the statistical temporal regularities between auditory and visual inputs, resulting in difficulties to use temporal coincidence as a reliable cue to integrate sensory information across modalities. Given that temporal proximity is one of the strongest of such cues in typically-developing populations but is less reliable in autistic individuals^4–6,17,33–40^, this may then result in decreased multisensory integration in this population.

**Figure 1:**
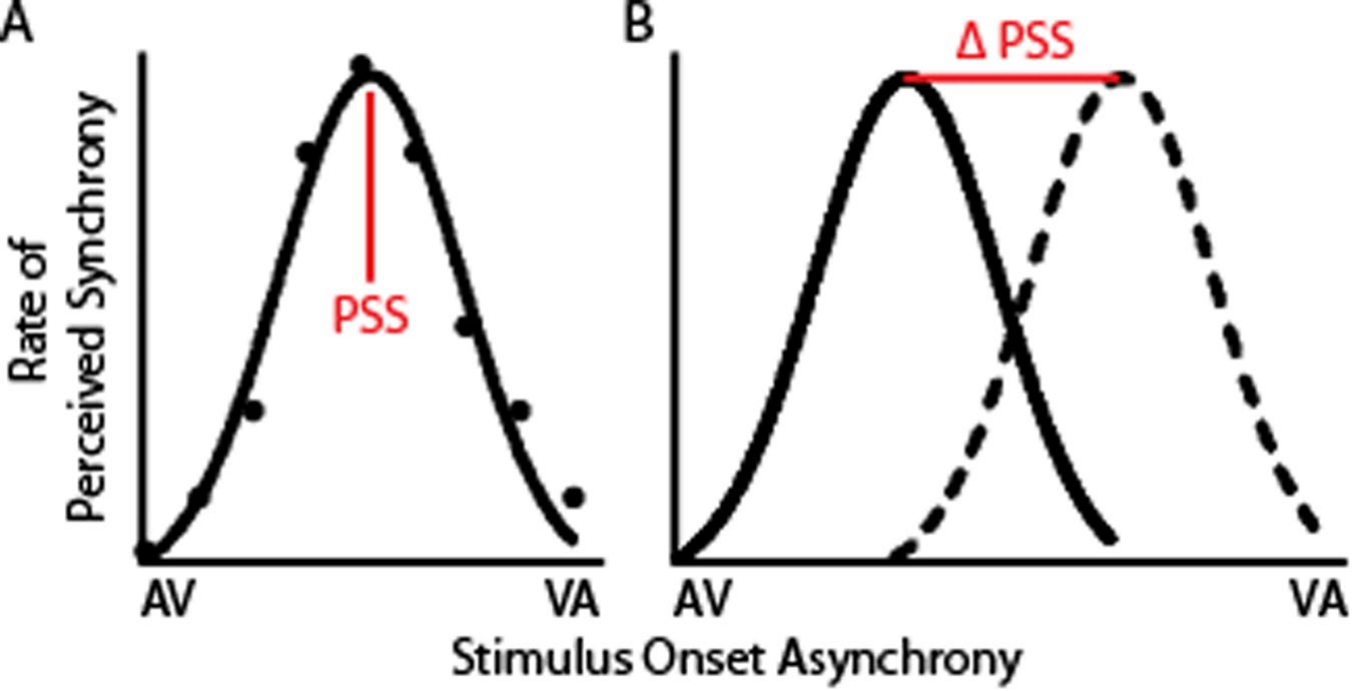
Temporal Recalibration. (**A**) The point of subjective simultaneity (PSS) is the temporal offset at which an individual is most likely to perceive an auditory and a visual sensory input as synchronous, and at which point an individual is most likely to integrate the auditory and visual information. AV stands for auditory input leading the visual input, VA stands for visual input leading auditory input. (**B**) When the statistical regularities of auditory and visual inputs are systematically altered, the point of subjective simultaneity adaptively shifts, an effect known as temporal recalibration. In this example, a participant was exposed to an asynchronous stimulus with visual information leading auditory information. As a result, a subsequent presentation with a visual lead is more likely to be perceived as synchronous than it would be if the initial stimulus was synchronous or auditory leading.

Statistical-learning paradigms have been developed to measure the ability to calibrate one’s perceptions of the world based on the statistical regularities of our environment, including audiovisual temporal statistics. Recent studies have shown that when an individual is immersed in an environment with altered temporal statistics, they adapt to these new statistical regularities in ways that reflect an updated probability map^48,49^. For example, if an individual is placed into an environment – even for just a few minutes – where visual sensory inputs consistently lead their corresponding auditory inputs, their subjective perception of synchrony will shift towards this new norm. This effect is called temporal recalibration (Fig. 1). This effect can even be seen more subtly in less immersive, trial-to-trial learning. When a participant is exposed to a single example of an asynchronous stimulus (e.g., visual information leading auditory information by 150 ms), a subsequent presentation with a visual lead is more likely to be perceived as synchronous than it would be if the initial stimulus was synchronous or auditory leading^50,51^. Rapid temporal recalibration has been preliminarily explored in ASD and these studies suggest that autistic children may indeed exhibit a decrease in statistical learning^52^. To our knowledge, however, the more immersive variants of these statistical learning paradigms have not been related to autistic symptomatology^48^.

**Figure 2:**
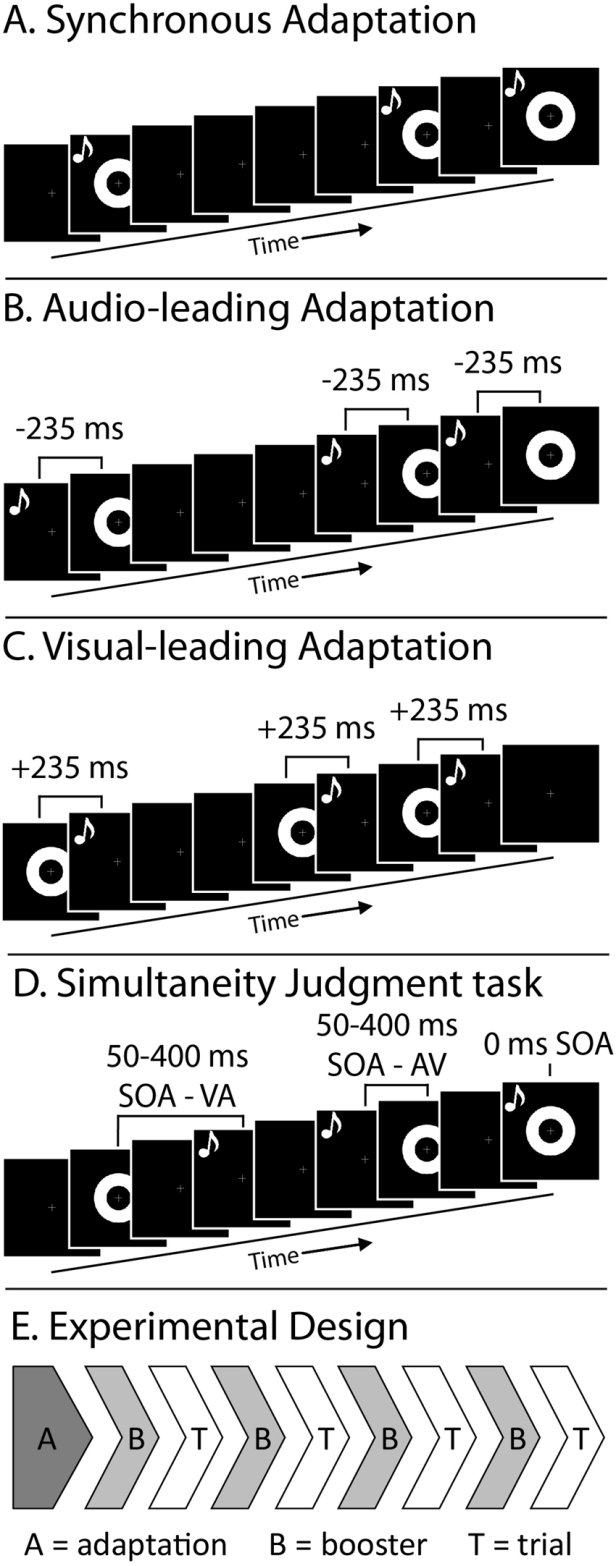
Experimental Design. Stimuli included visual flashes (rings) and auditory beeps (depicted by musical notes). Participants were exposed to three-minute adaptations of synchronous (**A**), consistent auditory-leading by 235ms (**B**), and consistent visual-leading by 235ms (**C**) presentations. Following each adaptation phase, participants completed a simultaneity judgement task with stimulus onset asynchronies (SOA) between the visual and auditory input ranging from −400 to +400 m, (**D**) interleaved with 10 s adaptation boosters identical to the adaptation phase (**E**).

Here, we take the next step in understanding the possible mechanistic role that statistical learning plays in decreased multisensory integration in autism, and explored the relationship between statistical learning and sub-clinical autistic traits in a non-clinical sample. Participants were exposed to three adaptation conditions: synchronous (SYNC, Fig. 2A), auditory leading (AV, Fig. 2B), and visual leading (VA, Fig. 2C). Following adaptations, participants completed a simultaneity judgement task (Fig. 2D) interleaved with adaptation “boosters” (Fig. 2E). Autistic traits were measured using the Autism Quotient (AQ)^53^, with a specific focus on the *Attention to Detail* subscale of the measure. High levels of attention to detail represent a cognitive style commonly associated with autism in which one attends more to fine-grained details of the world at the expense of more integrative, gestalt perceptions^54,55^. We hypothesized that individuals presenting with higher levels of autistic traits, and specifically a greater focus on local aspects of sensory inputs, will show weaker statistical learning abilities.

## Results

**Figure 3:**
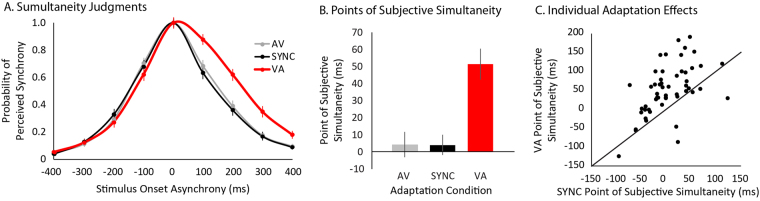
Multisensory Temporal Recalibration. Each individual’s perceptions of synchrony in a simultaneity judgment task following three distinct temporal adaptations (**A**) were used to calculate individual points of subjective simultaneity (**B**), the offset at which an individual was most likely to perceive a stimulus pair as synchronous – See Fig. 1A for a conceptual description. 48 of 54 participants showed positive adaptation effects following visual-leading adaptations (**C**), indicated by points being above and to the left of the unity line.

Each individual’s proportion of perceived synchrony (number of trials perceived synchronous relative to total number of trials) for each SOA in each adaptation condition was calculated (Fig. 3A). Gaussian curves were fit to each individual’s proportions of perceived synchrony. From these curves, the SOA at which an individual was most likely to perceive the presentations as synchronous was extracted for each condition (Fig. 3B; SYNC mean = 4.07 ms, SE = 5.95 ms; AV mean = 4.38 ms, SE = 7.35 ms; VA mean = 51.50 ms, SE = 8.87 ms), known as the Point of Subjective Simultaneity (PSS; Fig. 1A).

A repeated-measures ANOVA was conducted with respect to individual’s PSS, with adaptation condition as the within-subject factor (SYNC, AV, VA), revealing a significant main effect of adaptation condition (p < 0.001, F_(2,52)_ = 28.62, η_p_^2^ = 0.35). The assumption of sphericity was not violated (p = 0.38, χ^2^_(2)_ = 1.94). *Post Hoc*, pairwise t-tests with a Bonferroni correction revealed significant differences between the VA condition and the SYNC (p < 0.001, t = 6.23, d = 0.85) and AV (p < 0.001, t = 6.30, d = 0.79) conditions, but not between the SYNC and AV conditions (p = 0.96, t = 0.05, d = 0.01). Thus, a significant adaptation effect was observed in the VA condition but not in the AV condition, indicating that shifts in PSS were seen in the VA but not the AV adaptation condition.

**Figure 4:**
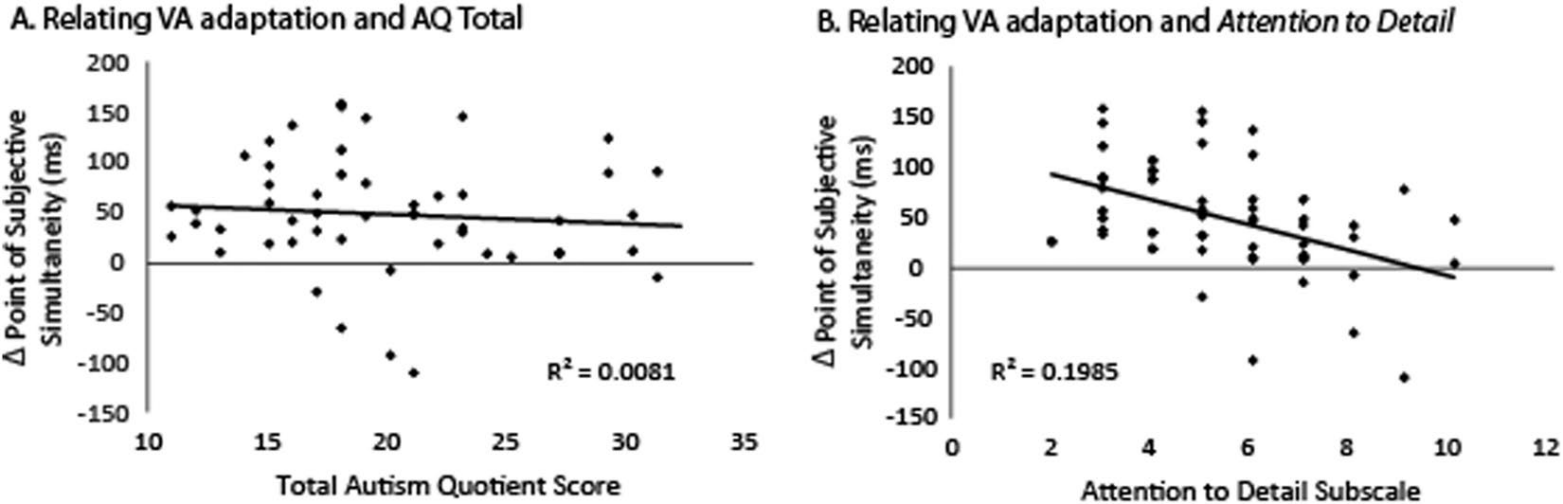
Relating Statistical Learning to Autistic Traits. The adaptation effect following the visual-leading adaptation phase did not significantly relate to overall Autism Quotient scores (**A**) but was significantly related to the Attention to Detail subscale (**B**).

Given the significant adaptation effect in the VA condition, a VA adaptation effect was calculated for each individual as the difference between the VA condition and the SYNC condition (mean = 47.43 ms, SE = 7.69 ms). To ensure that this effect was not driven by a minority of individuals showing a large effect, a binomial test was conducted testing for the proportion of individuals showing a shift towards a more visual-leading PSS, and 48 of 54 participants showed a positive shift (Fig. 3C; p < 0.000001). The VA adaptation effects were then correlated with individuals’ total AQ scores (Fig. 4A, p = 0.53, r = −0.09) and importantly, their *Attention to Detail* subscale scores (Fig. 4B, p = 0.001, r = −0.45), revealing that individuals with higher scores on the *Attention to Detail* subscale exhibited less temporal adaptation.

Exploratory analyses of VA adaptation to the other subscales of the AQ revealed no significant effects at either a Bonferroni corrected level (α = 0.0125) or an uncorrected level (*Social Skills*, p = 0.71, r = 0.05; *Attention Switching*, p = 0.44, r = 0.11; *Communication*, p = 0.40, r = −0.12; *Imagination*, p = 0.08, r = 0.25). Furthermore, the non-significant AV adaptation effect was not correlated with any AQ scales (Total, p = 0.70, r = −0.06; *Attention to Detail*, p = 0.42, r = −0.12; *Social Skills*, p = 0.64 r = −0.07; *Attention Switching*, p = 0.25, r = 0.16; *Communication*, p = 0.44, r = −0.11; *Imagination*, p = 0.97, r = 0.01).

## Discussion

Atypical sensory perception in autism has been widely reported, including changes in the manner in which autistic individuals integrate sensory information across sensory modalities. Here, we have demonstrated that these difficulties may be related to a decreased ability to make use of environmental statistical regularities that are commonly used to accurately associate sensory inputs across modalities. Specifically, the autistic trait of *Attention to Detail*, which captures the tendency to focus on individual pieces of information at the expense of perceiving more global, perceptual wholes, was associated with a decreased ability to update representational maps of environmental temporal statistics relative to a current sensory input. In short, the greater an individual’s focus on local aspects of sensory inputs, the less likely they were to recalibrate their perception of temporal synchrony. Such temporal synchrony perception is of specific interest, as it is one of the strongest cues to integrate information across the senses.

### Temporal recalibration and ASD traits

Throughout development, the temporal relationship between two incoming sensory inputs develops into a strong cue to bind these inputs, as individuals implicitly learn the temporal statistics of the environment surrounding them. Such cues lead individuals to integrate auditory and visual information that occur within a narrow window of time^20,32^, known as the temporal binding window^3,19,56–61^. This temporal window develops asymmetrically, reflecting the statistics of the environment where visual information reaches the retina prior to auditory information reaches the cochlea. As such, individuals are more tolerant of visual-leading sensory inputs^20,32^.

Statistical learning of the temporal regularities of the environment does not stop after early development, but perceptual systems instead remain quite plastic. Previous research has shown that exposure to artificial environments in which temporal regularities are systematically altered, as in the current study, results in temporal recalibration such that an individual’s perception of synchrony shifts towards the temporal regularities of this new environment^48,49^. In fact, such temporal recalibration can occur even on the timescale of single-trial recalibration. For example, when a single presentation of an audiovisual stimulus pair is presented asynchronously, such as with a visual lead, individuals are more likely to perceive a subsequent visual-leading trial as synchronous and less likely to perceive an auditory-leading trial as synchronous^50,51^.

One current theory of autism, within the predictive coding framework, postulates that an underlying factor impacting autism is an decreased ability to appropriately integrate prior, historical representation of the world and current sensory inputs^43–45,62,63^. Relevant to the current study, this hypothesis predicts that individuals on the spectrum are less able to utilize statistical temporal regularities in the environment to enable the use of temporal synchrony of two sensory inputs as a cue to bind. The data collected in this study support this hypothesis. Individuals that showed a high level of the autistic trait of *Attention to Detail*, which measures the tendency to default towards local over global processing, were less likely to adapt to the statistics of the artificial environment created in the adaptation phases of the current study, and thus showed weaker temporal recalibration.

To our knowledge, no similar studies relating autistic traits with temporal recalibration have been conducted. However, there has been one rapid recalibration study in ASD which reported atypical single-trial learning of audiovisual temporal perception^52^. In that study, autistic and TD children completed a simultaneity judgment task using the same simple flash-beep stimuli as used in the current experiment, and measured the difference in PSS based on whether the previous trial had been visual or auditory leading. Autistic children exhibited a decrease in rapid temporal recalibration relative to their TD peers with such basic stimuli, providing converging evidence that statistical learning may be impaired in autism.

An impairment in statistical learning of audiovisual temporal regularities in autism may have several downstream consequences, whether due to decreased prior or overweighting current inputs. First, acuity in audiovisual temporal perception, as previously stated, provides a strong cue to bind sensory information across the senses. Diminished acuity in audiovisual temporal perception impairs the ability to detect temporal regularities between events and may then impact multisensory integration abilities in autism. Indeed, numerous studies have shown impaired multisensory temporal perception in autism^40^, and likewise many have shown impaired multisensory integration^4–14^. Two such studies reported an explicit link between multisensory temporal perception in ASD and this ability to integrate audiovisual speech signals, where individuals with less precise temporal perception showed concomitant decreases in integration^6,17^. Furthermore, recent evidence has linked these decreases in multisensory temporal perception and audiovisual integration to decreased speech perception abilities in ASD^17^, suggesting that atypical sensory processing may directly affect social communication, one of the diagnostic criteria for ASD. It seems plausible then, that multisensory temporal processing in autistic children is predictive of speech perception abilities^17^, which may then map on to the Communication subscale of the AQ. This was not the case found here, though the items loading on to the Communication subscale focus on higher level communication (i.e. “I frequently find that I don’t know how to keep a conversation going.”) relative to speech perception. With that said, the failure here to find a significant relationship (p = 0.40) should not be mistaken for evidence that there is not relationship.

Given that temporal recalibration correlated with only the Attention to Detail subscale, it is worth discussing the relationship between symptoms related to ASD. A diagnosis of ASD requires not only a single diagnostic symptom, but multiple symptoms across two subdomains, social and communicative issues as well as restricted interests and repetitive behaviours (RRBs), with sensory issues falling into the latter category. While symptoms from both of these categories are necessary, factor analyses clustering the presence/severity of these symptoms revealed that RRBs, including sensory issues, strongly clustered together, but were only weakly associated with social and communicative issues^64^. It should be noted, however, that while *Attention to Detail* and multisensory perception are both particularly relevant to ASD, these two abilities may be mechanistically related beyond the realm of ASD, even in the broader phenotype. That is, being more focused on detailed attributes of the environment may lead to a decrease in perception or statistical relationships between items in the environment in general.

### Statistical learning in Autism

The current results present strong evidence for a decrease in temporal recalibration related to ASD traits. Temporal recalibration, however, is only one form of statistical learning. To date, there have been many accounts of statistical learning being impacted in ASD^43–45,47,65,66^, however, these results have been far from universal, with a number of studies also showing intact statistical learning^67,68^. These studies of statistical learning in autism have typically been discussed in Bayesian terms through the predictive coding framework, where it has been hypothesized that autistic individuals have difficulties incorporating prior information about the statistical regularities of the world with current, incoming sensory information^41,42^. This difficulty has been hypothesized to arise from a failure to develop a robust probabilistic representation of the world^43,44,50^ or that the current sensory input is given proportionately greater weight that the priors, resulting in prediction errors^46^.

Bayesian modeling of adaptation effects more generally has explored the differential effects that a flat prior probability map and a change in the weighting, or reliability, of incoming sensory information would have on such adaptation^69^. These models suggest that adaptation itself likely does not result from an update or prior probability maps, but instead that adaptation results from changes in reliability in the vicinity of the adapting stimulus, thus changing the likelihood probabilities. In autism the, it has been hypothesized that these representations of incoming sensory information and their associated prediction errors are *too* precise^46^. When prediction errors are overly precise, an individual sensory input is more likely to be heavily weighted, or treated as novel as opposed to a repetition of a previous experience. As a novel input, the sensory input is thus not compared to a prior probability map, and there is a subsequent failure to incorporate such prediction errors into generative prior probability maps. This conceptualization has been encapsulated in the High, Inflexible Precision of Prediction Errors in Autism (HIPPEA) model^46^.

While the HIPPEA model describes statistical learning in autism in general, a similar conceptualization has been formulated specifically related to multisensory temporal processing in autism^70^. It has been hypothesized that highly weighted prediction errors during temporal order judgments (“which came first”) or simultaneity judgments, as used in the current study may lead to wider TBWs in autism^4,6,39,40^, an effect that may be exacerbated by the nature of the task, where participants are explicitly directed to compare differences between the stimuli^71^. It should be noted that this concept has not been tested in terms of measuring standard neurological indices, such as increased beta-band activity^72^.

### Asymmetry in temporal recalibration

The finding that multisensory temporal perception can be recalibrated via adaptation after exposure to consistently asynchronous audiovisual presentations confirms previous reports of this effect^54,55^. These data also exhibited an asymmetrical effect of VA and AV adaptation, whereby VA adaptation induced a significant temporal recalibration but AV adaptation did not. Previous reports have shown numerically greater shifts with VA adaptations relative to synchronous exposure in similar paradigms^49^, and a study of perceptual learning using a feedback paradigm to narrow the temporal binding window has also concluded that the auditory-leading side of the window was not malleable^73^. Thus, there is converging evidence for asymmetrical plasticity of audiovisual temporal integration with a number of possible accounts. The first is based upon the fact that the relative timing of incoming auditory and visual sensory information is highly variable depending on the physical distance from the source – the further the source, the more visual leading the input becomes. Thus, the processing of visual-leading inputs may be more sensitive to adaptive recalibration. In a similar manner, auditory-leading sensory pairs are extremely rare relative to their visual-leading counterparts in the natural environment. Putting this in terms of Bayesian priors, the probability of an auditory stimulus input preceding a visual input that originates from the same source may be so heavily outweighted by prior experience that a three-minute exposure to AV adaptation may not be enough to overcome this prior weighting.

A separate account is derived from recent preliminary evidence suggesting that there may be distinct mechanisms underlying the integration of auditory- and visual-leading multisensory inputs^73^. In this study, participants underwent a perceptual-learning paradigm aimed at narrowing the temporal binding window^27,74–76^. Importantly, this study^73^ specifically attempted to manipulate the perception of synchrony on the left (AV) and the right (VA) side of the window independently. This study found that the auditory-leading side of the window was not malleable, and that visual-leading training only improved perception of visual-leading presentations and not auditory-leading presentations. Likewise, auditory-leading training did not improve perception of visual-leading stimuli. The current findings, that VA adaptation induces temporal recalibration while AV adaptation does not, provide further support for the possibility of separate mechanisms.

## Conclusions

This study was the first to our knowledge to report a relationship between statistical learning abilities and specific autistic traits. Individuals that showed high levels of *Attention to Detail* were likely to show weaker adaptations to the statistical temporal regularities of their environment. These data support the predictive coding hypothesis of autism, suggesting that a decreased reliance on previous perceptual experiences may be related to autism symptomatology. The finding that only adaptation to visual-leading presentations evoked temporal recalibration also provides evidence that the integration of auditory- and visual-leading multisensory inputs may in part be supported by distinct mechanisms.

## Methods

### Participants

A total of 60 undergraduate students from the University of Toronto participated in this study. Six participants were excluded from analyses because their results exceeded the sample’s average PSS by over three standard deviations, suggesting that they failed to follow the task instructions and properly complete the task, resulting in 54 participants (44 female, mean age = 19 years). All participants reported normal or normal-to-corrected vision and hearing, as well as absence of neurological disorders. All procedures were approved by the University of Toronto Research Ethics Board and conformed to the standards of the Canadian Tri-Council Research Ethics guidelines, and written, informed consent was obtained from all participants prior to participation.

### Procedure

#### Overview

Experimental designs were adapted from Fujisaki *et al*., 2004. All participants completed a simultaneity judgment (SJ) practice run, followed by three experimental conditions. Each experimental condition consisted of either a synchronous, auditory-leading, or visual-leading adaptation phase (Fig. 2A–C), followed by interleaved SJ trials and adaptation boosters (Fig. 2D,E). Experimental condition orders were counterbalanced across participants. Following behavioural testing, each participant completed the Autism Quotient (AQ)^53^ to assess autistic traits. All tasks were completed in light and sound controlled testing rooms. Participants sat at a fixed distance from a computer monitor (60 cm) and keyboard, stabilized by a chin rest. Participants were told to attend to the fixation throughout the study, and answer all questions as quickly and accurately as possible. Researchers monitored participant compliance with a live stream camera. All stimuli were presented in Matlab using the Psychtoolbox^77,78^.

#### Practice

Prior to experimental conditions, participants completed a practice SJ task. During the SJ practice, auditory and visual stimulus pairs were presented with stimulus onset asynchronies (SOA) that ranged from −400ms (audio-leading) to +400ms (visual-leading) in 100 ms increments, and participants were asked to report whether they perceived the two stimuli as occurring at the same time or at different times. If perceived at the same time, participants pressed ‘S’ on the keyboard; if different, they pressed ‘D’ on the keyboard. Six trials at each SOA were presented, amounting to 54 practice trails. Trial orders were randomized. Visual stimuli consisted of a white ring surrounding a fixation cross presented for 33 ms. Auditory stimuli consisted of a 1000 Hz pure-tone beep presented for 33 ms via sound cancelling headphones.

#### Adaptation

Each experimental condition began with a three-minute adaptation phase, during which participants were continually presented with audiovisual stimulus pairs with a fixed temporal relationship. In the synchronous condition (SYNC), audiovisual pairs were presented at the same time. In the visual-leading condition (VA), the visual stimulus onset preceded the auditory onset by 235 ms, and in the audio-leading condition (AV), the auditory stimulus onset preceded the visual onset by 235 ms. These offsets were chosen based on Fujisaki *et al.’s* 2004 study in which they reported that 235 ms offsets produced the strongest temporal recalibration. Stimuli were identical to those in the SJ task, except instead of a fixation cross, a fixation dot was presented centrally. The time interval between adjacent adaptation stimulus pairs was randomly jittered with a 776 ± 259 ms fixation in order to maximize stimulus density while avoiding unintended audiovisual grouping.

To ensure participants’ attentiveness during adaptation, they were instructed to complete an orthogonal deviant detection task. This involved pressing the spacebar whenever they observed either a smaller visual stimulus (two-thirds the original size during adaptation) or heard a lower-pitch beep (500 Hz). These deviants appeared on average once every 20 trials with random spacing. As such, participants were required to pay attention to both stimulus modalities.

#### Simultaneity judgment task

Following the three-minute adaptation exposure, participants completed SJ tasks identical to those in the practice run, interleaved with adaptation boosters. Boosters included ten seconds of adaptation identical to that in the adaptation phase. At the end of each booster, the fixation dot was replaced with a fixation cross, indicating that an SJ trial was beginning. The fixation cross with a randomly jittered duration ranging from between 1500 and 2500 s, followed by an SJ trial. The subsequent adaptation booster began immediately following participant response. Six trials at each SOA were presented for a total of 54 trials, with trial orders randomized. Each condition lasted for a duration of 15 minutes. For a graphical representation of each experimental condition’s format, see Fig. 3B.

#### Autistic traits

An online version of the AQ^53^ was administered after the behavioural portion of the experiment using Qualtrics. The AQ assesses five areas typically associated with autistic traits: social skills, attention switching, attention to detail, communication, and imagination. Each scale is represented by ten items, and each item is rated on a four-point Likert scale, with higher scores corresponding with increased ASD symptoms. The AQ has high internal consistency (Cronbach’s α = 0.97) and test-retest reliability (r = 0.85). Of particular interest here is the *Attention to Detail* subscale, a ten-item subscale with resultant scores ranging from 0–10. An example AQ item belonging to the *Attention to Detail* subscale is the following: “I usually concentrate more on the whole picture, rather than the small details.”

### Analysis

Analysis of the SJ task was modeled after Fujisaki *et al.’s* original 2004 work. For each individual and for each adaptation condition, mean rates of perceived synchrony were fitted with a Gaussian curve using the *fit* function in Matlab. The point of subjective simultaneity (PSS), or the point at which each individual, on each adaptation condition, was most likely to report a stimulus pair as synchronous, was then extracted from the fitted Gaussian. An initial, repeated measures ANOVA was conducted across the three adaptation conditions, followed by *post hoc* pairwise comparisons.

Adaptation effects for the AV and VA conditions were then calculated for each individual, as the shift in PSS seen with each condition relative to the PSS measured in the SYNC condition. Following any findings of significant temporal adaptation, these adaptation effects were then correlated with (1) participants’ *Attention to Detail* subscale of the AQ, and (2) participants’ overall AQ scores, with the hypothesis that individuals who exhibited high levels of autistic traits, and particularly in the *Attention to Detail* subscale, would show the lowest levels of adaptation.

Following these *a priori* analyses, exploratory analyses were conducted correlating significant adaptation effects to the remaining four subscales of the AQ. Bonferroni corrections were used for all post hoc analyses, here resulting in α = 0.0125.

### Data availability

The datasets generated during and/or analysed during the current study are available from the corresponding author on reasonable request.
